# Ankle blood pressure as a predictor of total and cardiovascular mortality

**DOI:** 10.1186/1471-2261-8-3

**Published:** 2008-02-12

**Authors:** Heikki Hietanen, Rauni Pääkkönen, Veikko Salomaa

**Affiliations:** 1 Helsinki Deaconess Institute, Alppikatu 2, Helsinki, Finland; 2 KTL-National Public Health Institute, Mannerheimintie 166, FI-00300 Helsinki, Finland

## Abstract

**Background:**

The ankle blood pressure is commonly used as a ratio to the brachial blood pressure, called ankle-brachial index (ABI). Very few studies have considered the independent value of the ankle blood pressure without indexing it to the brachial blood pressure. We examined the value of ankle blood pressure, together with the exercise blood pressure, as a predictor of cardiovascular (CVD) and total mortality.

**Methods:**

A prospective follow-up study of 3,858 consecutive ambulatory patients (mean age 51 years, 65,9% male) referred to a symptom-limited exercise test between August 1989 and December 1995. The cohort was followed up for all-cause and CVD mortality until December 31, 2004, by record linkage with the National Causes-of-Death Register. The independent value of ankle blood pressure as a predictor of cardiovascular and total mortality was assessed using Cox proportional hazards modelling.

**Results:**

The average follow-up time was 14 years, during which 346 persons died, 108 of them due to CVD. Persons with normal (<140 mmHg) resting brachial blood pressure, ankle blood pressure < 175 mmHg and exercise blood pressure at moderate exercise level ≤215 mmHg at baseline investigation, had the best prognosis and were taken as the reference category. Among persons with elevated ankle blood pressure (≥175 mmHg) but normal or borderline resting brachial pressure and normal exercise blood pressure (≤215 mmHg) at moderate exercise level the multivariate-adjusted hazard ratios (HR, 95% confidence interval) for CVD and total mortality were 2.70 (1.52 – 4.80) and 2.13 (1.58 – 2.85), respectively. Similar and equally significant HRs were observed in persons with both elevated ankle blood pressure and elevated exercise blood pressure, as well as in those persons with elevated exercise blood pressure but ankle blood pressure < 175 mmHg.

**Conclusion:**

These results suggest that the ankle blood pressure has an independent value as a marker of arterial stiffness or subclinical atherosclerosis and a risk of future mortality in middle-aged, asymptomatic persons.

## Background

There are several established procedures for estimating subclinical atherosclerotic changes in human arteries. Increased carotid intima-media thickness is associated with future cerebrovascular and cardiovascular events [1,2]. Pulse wave velocity is related to arterial wall stiffness, future hypertension and cardiovascular diseases [3,4]. Coronary artery calcium screening is the latest method for evaluating the CVD risk in asymptomatic patients [5,6]. All these methods, however, require sophisticated equipment and a specialized user. Accordingly, they are not well suitable for the screening of early arterial changes in a routine office practice and there is a great need for a simple non-invasive tool that could be used in an office setting to screen for arterial stiffness or subclinical atherosclerosis.

The ankle blood pressure is usually measured in conjunction with the arm blood pressure and the ankle-brachial pressure index (ABI) is calculated. Decreased index is strongly associated with cardiovascular diseases [7-16]. Also elevated ABI value seems to be a significant risk factor of CVD [17,18].

Patients with exaggerated exercise blood pressure reaction have increased cardiovascular disease morbidity compared with those with normal exercise blood pressure reaction [19-22]. It is explained by increased sympathetic tone and structural changes in arteries.

We hypothesized that in the beginning of the arterial stiffening and atherosclerosis the ankle blood pressure may be determined by local factors only, i.e., blood pressure and the elastic properties of arteries. The elevated ankle blood pressure might be one of the earliest signs of adverse changes in the cardiovascular system. Stenotic changes with abnormal ABI are fairly seldom seen in middle-aged persons [13,14]. Ankle blood pressure ≤ 175 mmHg but exaggerated elevation of brachial systolic blood pressure at a moderate exercise level reveals those patients in whom stenotic changes along the conduit vessels decrease the ankle blood pressure and this group has to be considered as a separate entity.

Long-term prospective studies on the value of ankle blood pressure in middle-age persons are currently sparse. Therefore, the aim of the present study was to assess the independent value of ankle blood pressure, together with the brachial exercise blood pressure, as a predictor of cardiovascular and total mortality during the average follow-up of 14 years.

## Methods

### Study population

Subjects for this investigation were derived from a group of 4,038 consecutive ambulatory patients, who underwent symptom-limited bicycle exercise test at the Helsinki Deaconess Institute between August 1989 and December 1995. The patients were referred by occupational health physicians to a symptom-limited exercise test to rule out coronary heart disease and evaluate physical fitness. More precisely, 1,734 were sent for the evaluation of physical fitness, 1,799 for diagnostic testing due to chest pain or shortness of breath, 488 because of suspected arrhythmias and 17 for mixed reasons, mainly for suspicion of exercise-induced asthma. Patients with a history of myocardial infarction, percutaneous coronary angioplasty, coronary artery bypass grafting, congestive heart failure or stroke, were excluded from the analysis. The final study group consisted of 3,858 patients. The study was approved by the Ethical Committee of the National Public Health Institute.

### Baseline vascular examination

Brachial blood pressure was measured by trained technicians using the auscultatory method with a standard sphygmomanometer from the left arm of the subject in a supine position after a 5 minute rest. The ankle blood pressure was simultaneously measured from the right leg using a Doppler probe with a mercury sphygmomanometer. If the pulse of the posterior tibial artery was absent, the ankle blood pressure measurements were taken on the dorsalis pedis artery.

### Exercise ECG testing and measurement of exercise blood pressure

Exercise testing was conducted on an electronically braked bicycle. The starting load was 50 Watts for men and 40 Watts for women, and the load was increased every 3 minutes by 50 Watts for men and 40 Watts for women. Blood pressure was measured with a sphygmomanometer at 2 minutes at all loads and immediately prior to test termination. Readings were recorded to the nearest 5 mmHg. The 2-minute blood pressure recording at the moderate exercise level (150 W for men and 120 W for women) was used in the analyses. If that level was not reached, blood pressure from the lower level was used. The test was continued until a subject refused to continue, or until the attending physician felt it unsafe to continue. The criteria for myocardial ischaemia during the exercise test were ischaemic changes in ECG defined as ST depression >1.0 mm at 60 ms after the J-point with typical ischaemic complaints.

### Other baseline characteristics

Other risk factors assessed at baseline were age, gender, body mass index (BMI), smoking status, medical history, parental history of early cardiovascular disease, physical working capacity, self-reported history of cardiovascular diseases, total cholesterol and glucose.

### Blood pressure groups

Subjects were divided into five groups based on resting ankle and exercise blood pressure at the moderate exercise level (men 150 Watt, women 80 Watt): 1) Reference group, where the resting ankle blood pressure was < 175 mmHg and the exercise blood pressure ≤ 215 mmHg; 2) patients with elevated ankle blood pressure (≥175 mmHg) but normal exercise blood pressure (≤215 mmHg); 3) patients with elevated ankle (≥175 mmHg) and elevated exercise blood pressure (>215 mmHg); 4) elevated exercise blood pressure (>215 mmHg), but ankle blood pressure <175 mmHg; and 5) patients who could not be classified. Group 4 consists of patients with a discrepancy between the ankle blood pressure and brachial exercise systolic blood pressure, which indicated significant stenotic changes along the conduit vessels. The unclassifiable group could not reach the moderate exercise level because of a specified reason (for example ischaemic heart disease) or an unspecific reason (for example poor physical fitness).

### Follow-up procedures

The mortality follow-up data were available up to 15 years (range 12 – 15 years) after the exercise test, until December 31st, 2004. Deaths were ascertained by record linkage of the study data to the National Causes-of-Death Register, on the basis of the personal identification code unique to every resident of Finland. Thanks to the country-wide register, the coverage of the follow-up was 100%.

In the Causes-of-Death Register deaths were coded according to the Ninth (until Dec. 31st, 1995) and Tenth (since the beginning of 1996) versions of the International Classification of Diseases (ICD). The primary endpoint was cardiovascular death, and all-cause mortality was used as a secondary endpoint. ICD-9 codes 410 to 414, 431, 436, 798, 4330A, 4331A, 3339A, 4341A, 4349A, 4376A or ICD-10 codes I20–I25, I46, I61, I63–I64, R96, R98 as the underlying cause of death were taken as cardiovascular deaths. Altogether, 346 persons died during the follow-up, 108 of the deaths were cardiovascular. As a whole, the study consisted of 52,234 person-years of follow-up.

### Statistical methods

Data are expressed as mean ± SD for continuous variables, or counts and proportions for categorical variables. The following cardiovascular risk factors were dichotomized: early parental cardiovascular death (yes or no), self-reported elevated cholesterol (>6 mmol/l, yes or no), self-reported elevated blood glucose (>6 mmol/l, yes or no) and current smoking (yes or no). Age, BMI, smoking (years, packet/day) and blood pressure (mmHg) were handled as continuous variables.

Continuous variables were compared between the blood pressure groups with analysis of variance and proportions with chi-square tests. Univariate associations between different blood pressure indicators were evaluated with Pearson's product-moment correlation coefficients.

Associations between the blood pressure groups and mortality were analyzed using Kaplan-Meier survival curves and log-rank tests. Cox proportional hazard models were used for estimating the multivariate-adjusted independent associations of the blood pressure groups with total and cardiovascular mortality. Results were expressed as hazard ratios (HR) and 95% confidence intervals (CI) compared to the reference group. The basic models were adjusted for age and sex. The larger models were further adjusted for BMI, physical working capacity (metabolic equivalents = METs), self-reported blood glucose and cholesterol, current smoking and early parental history of cardiovascular disease. The statistical analyses were carried out with R (Version 2.3.1).

## Results

The mean age at baseline was 50.5 ± 10.0 years (range 15 – 84), and the mean BMI was 26.1 ± 3.8 kg/m2. The mean brachial blood pressure was 133.1 ± 18.7/85.3 ± 10.9 mmHg) and pulse 73.8 ± 12.9/min. The correlation coefficient between the brachial blood pressure at rest and the exercise blood pressure at the moderate exercise level (men 150 Watts, women 80 Watts) was 0.548 (n = 2,313, P < 0.0001) in men and 0.649 (n = 1,211, P < 0.0001) in women. The correlation coefficient between the ankle blood pressure and systolic blood pressure at the moderate exercise level was 0.543 (P < 0.0001) in men and 0.597 (P < 0.0001) in women.

Table 1 compares cardiovascular risk factors in different blood pressure categories at baseline. The ABI differed significantly between the groups being highest in groups 2 and 3 and lowest in groups 4 and 5. The reference group (n = 2,203) was younger and leaner. In this group all risk factors were more favourable compared with the other groups. Myocardial ischaemia during exercise was diagnosed in 94 patients. The all-cause mortality during follow-up was 89 patients (4.0%), 20 (0.9%) due to cardiovascular causes.

**Table 1:** Characteristics of the Study Participants by Ankle Blood Pressure (ABP) and Exercise Blood Pressure (EBP) Group.

	Normal ABP Normal EBP	Elevated ABP and Normal EBP	Elevated ABP and Elevated EBP	Normal ABP and Elevated EBP	Unclassified	
	n = 2203	n = 791	n = 509	n = 222	n = 133	p
Age (years)	47 (10)	55 (9)	54 (7)	51 (9)	59 (10)	0.001*
Male/female	1419/820	447/358	435/87	205/21	105/30	0.001†
Body mass index (kg/m^2^)	25.4(4)	26.8 (4)	27.5 (4)	26.9 (4)	25.7 (4)	0.001*
Syst. BP (mmHg)	124 (14)	144 (17)	152 (17)	135 (16)	137 (20)	0.001*
Diast. BP (mmHg)	82 (9)	90 (10)	94 (10)	85 (9)	85 (11)	0.001*
Pulse/min	73 (13)	75 (13)	75 (13)	74 (13)	75 (14)	0.001*
SBP80 (women)	172 (20)	192 (15)	229 (11)	233 (13)	-	0.001*
SBP150 (men)	186 (18)	199 (14)	236 (16)	231 (12)	-	0.001*
SBPMax	193	203	240	236	178.3	0.001*
Ankle blood pressure (mmHg)	150 (17)	194 (14)	198 (18)	157 (14)	141 (37)	0.001*
ABI	1.21 (0.38)	1.31 (0.14)	1.27 (0.17)	1.16 (0.14)	1.03 (0.27)	0.001*
ABI < 0.97 n (%)	66 (3)	0 (0)	0 (0)	22 (1)	39 (29)	0.001†
Self-reported abnormal total cholesterol n (%)	925 (42)	340 (43)	234 (46)	122 (55)	74 (56)	0.001†
Self-reported abnormal glucose n (%)	132 (6)	95 (12)	66 (13)	27 (12)	35 (26)	0.001†
Pos. family history n (%)	793 (36)	324 (41)	188 (37)	95 (43)	69 (529	0.005†
Pack-years of smoking	9 (11)	9 (12)	13 (14)	14 (13)	20 (16)	0.001*
Current smokers n (%)	308 (14)	71 (9)	71 (14)	36 (16)	51 (38)	0.001†
METs	8.4 (3)	6.6 (2)	7.4 (2)	7.9 (1.9)	4.6 (1.5)	0.001*
Cardiovascular medication n (%)	22 (1)	285 (36)	178 (35)	33 (15)	60 (45)	0.001†
Diagnosed ischemic heart disease n (%)	88 (4)	142 (18)	46 (9)	13 (6)	72 (54)	0.001†
Deaths during follow-up n (%)	88 (4)	119 (15)	56 (11)	22 (10)	60 (45)	0.001†
Cardiovascular deaths during follow-up n (%)	20 (0.9)	40 (5)	20 (4)	9 (4)	23 (17)	0.001†

Mean values (S.D.) or proportions (%), * ANOVA, † χ^2 ^test

SBP80 = systolic blood pressure at the exercise level of 80 Watts

SBP150 = systolic blood pressure at the exercise level of 150 Watts

SBPMax = maximum systolic blood pressure during exercise

ABI = Ankle Brachial Index

METs = Exercise capacity measured in metabolic equivalents

In patients (n = 791) with elevated ankle blood pressure (≥175 mmHg) and normal exercise blood pressure the resting brachial blood pressure was normal or slightly elevated: 144 ± 17/90 ± 10 mmHg. Of this group 36% were on cardiovascular medication. In patients without any medication (n = 510) the blood pressure was normal or slightly elevated: 142.2 ± 15/90.0 ± 10.1 mmHg. In this whole group, 116 (14.7%) patients died during the follow-up period, 37 (4.7%) due to cardiovascular causes.

Among patients (n = 509) with elevated ankle blood pressure and elevated exercise blood pressure the male sex dominated. The brachial blood pressure at rest was elevated: 152.8 ± 17/94.0 ± 10 mmHg. Compared with the previous group, BMI and ankle blood pressure were also higher and the smoking history was longer. In patients without any medication (n = 348) the mean blood pressure at rest was elevated: 150.6 ± 16/93.1 ± 10 mmHg. Myocardial ischaemia during exercise was diagnosed in 44 (8.6%) patients. The all-cause mortality in this group was 58 patients (11.4%) and the mortality due to cardiovascular disease was 20 patients (3.9%).

In patients with the discrepancy between the ankle blood pressure and systolic blood pressure (group 4, n = 222) there was no correlation between the ankle blood pressure and exercise blood pressure at moderate level (r = 0.050, n = 201, p = 0.49) among men and r = 0.059, n = 21, p = 0.80 among women). Compared with the reference group the ankle blood pressure was a bit higher. The male sex dominated (201/222) in this group, and smoking was more common than in groups 1 and 2. Fifteen per cent were on cardiovascular medication. Myocardial ischaemia was diagnosed in 14 patients (6.3%), two of them died during the follow-up. The all-cause mortality was 22 patients (9.9%) and the mortality due to cardiovascular causes was 8 (3.6%).

The fifth group (n = 133) was older. They could not tolerate moderate exercise level because of ischaemic heart disease (n = 71) or leg atherosclerotic disease (ABI <0.97, n = 38). Abnormal lung function was observed in 10 patients (oxygen <90%). Almost half of this group (45.2%) died during the follow-up period, 23 (17.3%) due to cardiovascular causes. There were 43 patients with low fitness without any specific reason. Twenty (47%) of them died during the follow-up period, 6 (14%) due to cardiovascular causes. They tended to be current (n = 19) or ex-smokers (n = 7). The male sex dominated (36/43). Nine were on antihypertensive medication.

**Figure 1:**
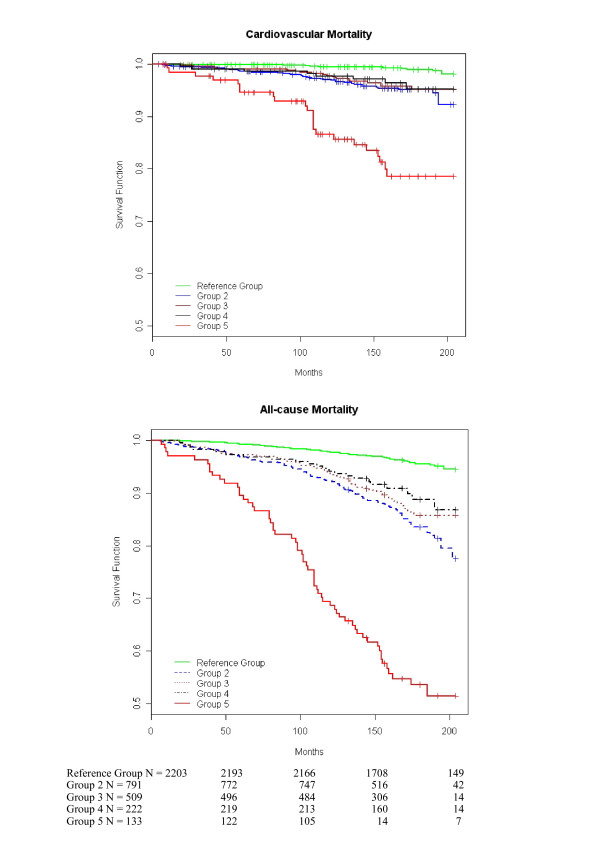
**Cardiovascular and all-cause mortality by the blood pressure group**. Kaplan-Meier survival curves for cardiovascular and all-cause mortality. Log-rank test for the survival difference between the blood pressure groups: p < 0.0001 for cardiovascular mortality and p < 0.0001 for all-cause mortality. The numbers indicate people remaining in the follow-up at different points in time. All participants were not followed up for 200 months. The reference group consists of patients with normal brachial blood pressure (< 140 mmHg), ankle blood pressure <175 mmHg and exercise blood pressure ≤215 mmHg at the moderate exercise level. Group 2 had elevated ankle blood pressure (≥175 mmHg) but normal exercise blood pressure. Group 3 had elevated ankle and exercise blood pressure. Group 4 had ankle blood pressure < 175 mmHg but elevated exercise blood pressure (discrepancy) and group 5 could not be classified because of poor exercise tolerance.

Figure 1 shows the Kaplan-Meier survival curves for cardiovascular and all-cause mortality in different blood pressure groups. For both endpoints the curves diverge continuously and significantly throughout the 14 years of follow-up.

Compared with group 1, the age and sex-adjusted hazard ratios of CVD and all-cause mortality were significantly elevated in all other groups (Table 2). Further adjustment for traditional risk factors, such as smoking, BMI, parental history of early CVD, self-reported elevated cholesterol and blood glucose attenuated the HRs slightly, but they nevertheless remained clearly significant.

**Table 2:** Hazard Ratios (HR, 95% Confidence Interval (CI)) of Cardiovascular and All-Cause Deaths According to Specified Blood Pressure Groups.

	Model 1			Model 2		
	HR	95% CI	p	HR	95% CI	p

Cardiovascular death						
Group 1	1	(reference)		1	(reference)	
Group 2	3.11	1.79 – 5.41	< 0.0001	2.62	1.49 – 4.62	0.0003
Group 3	2.77	1.48 – 5.16	0.003	2.48	1.33 – 6.62	0.003
Group 4	2.78	1.22 – 6.33	0.02	2.36	1.04 – 5.37	0.02
Group 5	8.45	4.42 – 16.15	<0.0001	4.28	2.07 – 8.87	<0.0001
						
All-cause death						
Group 1	1	(reference)		1	(reference)	
Group 2	2.40	1.81 – 3.20	<0.0001	2.13	1.59 – 2.85	< 0.0001
Group 3	2.01	1.43 – 2.81	<0.0001	1.80	1.29 – 2.53	<0.0001
Group 4	1.98	1.25 – 3.15	0.003	1.70	1.07 – 2.70	0.03
Group 5	5.92	4.15 – 8.46	<0.0001	3.21	2.15 – 4.79	<0.0001

Model 1: adjusted for age and sex

Model 2: adjusted for age, sex, BMI, smoking, early parental cardiovascular disease, exercise capacity measured in metabolic equivalents, self-reported elevated cholesterol and abnormal blood glucose

Please see Table 1 and the Methods-section for the explanation for the blood pressure groups

For comparison, we calculated the HRs for the different levels of ankle blood pressure alone (Table 3). Patients with abnormal ABI (<0.97) were kept as their own group. The age and gender adjusted HRs increased linearly with higher ankle blood pressure and were highest in patients with abnormal ABI. Multivariate-adjusted HRs behaved similarly, although they reached statistical significance for all-cause mortality only. Moreover, we calculated the HRs for the usual resting brachial systolic blood pressure, dichotomized at 160 mmHg (<160 mmHg vs ≥160 mmHg). In the age and sex-adjusted model, the HR for CVD death was 1.94 (1.23 – 3.06, p = 0.004). In the larger model the HR was attenuated to 1.6 (1.01 – 2.54, p = 0.04).

**Table 3:** Hazard Ratios (HR, 95% Confidence Interval (CI) of Cardiovascular and All-Cause Deaths in Different Levels of Ankle Blood Pressure (ABP).^1^

	Model 1			Model 2		
Ankle Blood Pressure Category (n)	HR	95% CI	p	HR	95% CI	p

Cardiovascular death						
100–150 mmHg (n = 1214)	1	(reference)		1	(reference)	
151–174 mmHg (n = 1082)	1.54	0.85 – 2.79	0.2	1.52	0.83 – 2.78	0.2
175–200 mmHg (n = 1086)	1.71	0.97 – 3.02	0.06	1.73	0.97 – 3.07	0.06
201–300 mmHg (n = 355)	1.98	1.03 – 3.84	0.04	1.77	0.91 – 3.45	0.1
Abnormal (< 0.97) ABI (n = 121)	2.38	1.10 – 5.17	0.03	1.53	0.68 – 3.4	0.3
						
All-cause death						
100–150 mmHg (n = 1214)	1	(reference)		1	(reference)	
151–174 mmHg (n = 1082)	1.30	0.95 – 1.79	0.1	1.30	0.94 – 1.79	0.2
175–200 mmHg (n = 1086)	1.44	1.06 – 1.95	0.02	1.47	1.08 – 2.00	0.02
201–300 mmHg (n = 335)	1.70	1.18 – 2.43	0.003	1.53	1.06 – 2.21	0.02
Abnormal (0.97) ABI (n = 121)	2.20	1.43 – 3.40	< 0.0001	1.49	1.00 – 2.33	0.02

Model 1: adjusted for age and sex

Model 2: adjusted for age, sex, BMI, smoking, early parental cardiovascular disease, exercise capacity measured in metabolic equivalents, self-reported elevated cholesterol and abnormal blood glucose

Please see Table 1 and the Methods-section for the explanation for the blood pressure groups

^1 ^Persons with low ABI separated as their own group

## Discussion

In the present study, we demonstrated that the ankle blood pressure gives us important information about the status of the arterial tree in middle-aged asymptomatic individuals. The main finding was that even those persons among whom the elevated ankle blood pressure was the only abnormal finding had 2.7-fold higher multivariate-adjusted risk of CDV death and 2.1-fold higher risk of death from any cause than persons with normal brachial, ankle and exercise blood pressures. This suggests that the measurement of ankle blood pressure could be a relatively simple, inexpensive and non-invasive tool for assessing early, subclinical atherosclerotic changes in young and middle-aged individuals. This finding is consistent with earlier studies suggesting a J-shaped association between ABI and CVD risk [5,17,18].

At least three independent lower-extremity large-vessel characteristics determine the ankle blood pressure: local pressure, elasticity of the vessels and the pulse wave. Arterial stiffness with increased pulse wave velocity and augmented pulse pressure cause a strong cyclic stretching on vascular smooth muscle cells [23-26]. Hypertrophy and structural changes decrease the elasticity of the conduit vessels, and elevated ankle blood pressure is measured. An unanswered question is, when is the ankle blood pressure determined only by local factors, and when do stenotic changes along the conduit vessels begin to have an effect on the peripheral ankle blood pressure. We postulate that this stage is reached when the exercise causes an exaggerated blood pressure reaction but the ankle blood pressure is within the normal limits.

Although the importance of exercise hypertension remains controversial, the majority of recent studies show that an exaggerated systolic blood pressure response to exercise is an independent predictor of future hypertension and CVD mortality [18-20,26-29]. It is associated with insulin resistance [30], hypercholesterolemia [31], carotid atherosclerosis [22] and other target-organ damage [32]. The pathophysiology of exaggerated blood pressure response is not yet fully understood. Two processes have been involved in most studies: increased sympathetic tone and structural changes in the vessels.

The categorization of our study cohort into five subgroups enabled a logical understanding of the chronological sequence of adverse changes in the ankle blood pressure. In patients with normal ankle, arm and exercise blood pressure, both CVD and total mortality during the follow-up were low even among persons with an abnormal exercise test. The elevated ankle blood pressure either with or without elevated exercise blood pressure was significantly associated with CVD mortality and all-cause mortality. It is associated with abnormally high ABI, which has also been found in other studies [17,18].

Patients with elevated exercise blood pressure but ankle blood pressure ≥175 mmHg are a more discrete group. With our cut-points (ankle blood pressure ≥175 mmHg and exercise blood pressure at moderate exercise level > 215 mmHg) they formed a group in which CVD and all-cause mortality were at the same level as in patients with elevated ankle blood pressure. The discrepancy between the ankle and exercise blood pressures can be explained by hemodynamically significant stenotic changes along the conduit vessels. The poor correlation between the exercise brachial blood pressure and the ankle blood pressure is in accordance with the existence of stenotic changes. Also, abnormally low ABI was observed in group 4 compared with the reference group.

Normally there is a strong correlation between the arm, ankle and exercise blood pressures as long as the stenotic changes along the conduit vessels are minor. Similar correlation between the systolic blood pressure and maximal or submaximal exercise blood pressure has been found in other studies [27]. Elevated ankle blood pressure alone is also associated with an increased risk for CVD- and overall mortality. Exercise test reveals further those patients, where changes in the conduit vessels decrease the ankle blood pressure and the healthy reference group becomes more accurately defined.

Low cardiovascular fitness is associated with premature mortality. The literature is filled with long-term follow-up studies, conducted in relatively healthy populations [33-36], or focused on clinical patient populations [36], which have indicated, that exercise capacity is a more powerful predictor of mortality than the other established risk factors. One third of our unclassifiable group died during the follow-up and most of them belonged to the group with low physical fitness without any specific reason.

Our study has certain limitations. The patients were referred by occupational health physicians and thus they do not represent a random sample of the general population. The reference group is a predominantly well-educated group. The ankle blood pressure was measured from one leg only. The blood glucose and total cholesterol were self-reported and only half of the patients knew their glucose value. However, our purpose was not to explain the aetiology of elevated ankle blood pressure but to examine its prognostic value. These limitations of our study should not affect the validity of the findings. Furthermore, most patients with a diagnosed MI during the follow-up had received pharmacological therapy or undergone invasive therapeutic procedures and their lifestyle had changed. Such a bias has probably led to an underestimation, rather than an overestimation, of the prognostic significance of elevated ankle blood pressure.

## Conclusion

In the present study we showed that the ankle blood pressure – without indexing to arm blood pressure – has an independent value as a marker of subclinical atherosclerosis in asymptomatic middle-aged patients. The elevated ankle blood pressure is one of the earliest sign of the adverse changes in the arteries. It identifies high-risk individuals and may provide the necessary motivation to promote lifestyle changes. On the other hand, exaggerated exercise blood pressure reaction with normal ankle blood pressure reveals those patients with stenotic changes along the conduit vessels. They are likely to need more detailed investigations and intensive therapy. Thus, measurement of the ankle blood pressure could be an inexpensive and non-invasive tool which helps to assess the CVD risk and to guide the intensity of other examinations and therapies.

## Competing interests

The author(s) declare that they have no competing interests.

## Authors' contributions

HH: Conceived the idea of the study, investigated the patients, carried out part of the statistical analyses and wrote the first draft of the manuscript.

RP: Took care of the data management and part of the statistical analyses. Contributed to the interpretation of the results and commented on the manuscript with important intellectual content.

VS: Supervised the study. Contributed to the follow-up of the patients and interpretation of the statistical analyses. Commented on the manuscript with important intellectual content.

All authors have read and approved the final manuscript.

## Pre-publication history

The pre-publication history for this paper can be accessed here:


